# Soap, water, and severe acute respiratory syndrome coronavirus 2 (SARS-CoV-2): an ancient handwashing strategy for preventing dissemination of a novel virus

**DOI:** 10.7717/peerj.12041

**Published:** 2021-09-17

**Authors:** M. Khalid Ijaz, Raymond W. Nims, Sarah de Szalay, Joseph R. Rubino

**Affiliations:** 1 Global Research & Development for Lysol and Dettol, Reckitt Benckiser LLC, Montvale, New Jersey, United States; 2 Department of Biology, Medgar Evers College of the City University of New York (CUNY), Brooklyn, New York, United States; 3 RMC Pharmaceutical Solutions, Inc., Longmont, Colorado, United States

**Keywords:** SARS-CoV-2, Hierarchy of susceptibility to inactivation, Infection prevention and control, Hand disinfection, Hand sanitizers, Anti-infective agents, Soaps, Virus inactivation, Virus removal, Enveloped viruses

## Abstract

Public Health Agencies worldwide (World Health Organization, United States Centers for Disease Prevention & Control, Chinese Center for Disease Control and Prevention, European Centre for Disease Prevention and Control, etc.) are recommending hand washing with soap and water for preventing the dissemination of severe acute respiratory syndrome coronavirus 2 (SARS-CoV-2) infections. In this review, we have discussed the mechanisms of decontamination by soap and water (involving both removal and inactivation), described the contribution of the various components of formulated soaps to performance as cleansers and to pathogen inactivation, explained why adherence to recommended contact times is critical, evaluated the possible contribution of water temperature to inactivation, discussed the advantages of antimicrobial soaps *vs*. basic soaps, discussed the differences between use of soap and water *vs*. alcohol-based hand sanitizers for hand decontamination, and evaluated the limitations and advantages of different methods of drying hands following washing. While the paper emphasizes data applicable to SARS-CoV-2, the topics discussed are germane to most emerging and re-emerging enveloped and non-enveloped viruses and many other pathogen types.

## Introduction

Personal hand hygiene, and hygiene in general, have played an integral role in several religious and cultural norms within different societies for centuries. Hand washing with soap is routinely employed as part of personal hygiene during everyday life in developed countries (World Health Organization, 2009). However, the important role played by contaminated hands in dissemination of infectious agents has only been realized since the 19^th^ century (Semmelweis, 1861).

**Figure 1:**
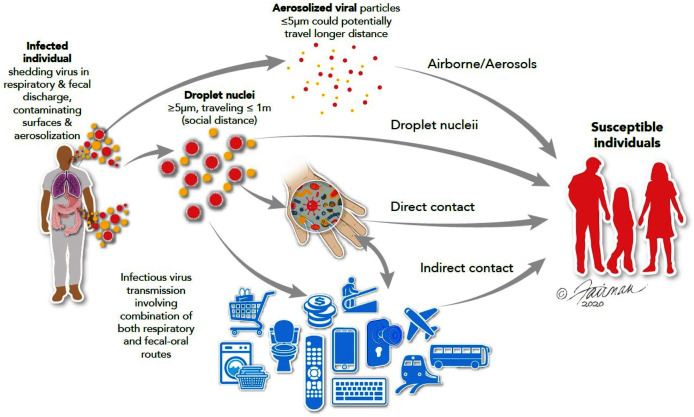
Routes of transmission of respiratory and enteric viruses, such as influenza, MERS-CoV, and SARS-CoV-2 (from Ijaz et al., 2020a). Illustrations by: Jennifer Fairman, © 2021, Fairman Studios, LLC.

The interventional role of hygiene agents in disrupting the chain of infection of pathogens is now well established (World Health Organization, 2009; Alum, Rubino & Ijaz, 2010; Stephens et al., 2019; Scott et al., 2020; Scott, Bruning & Ijaz, 2021). Many infectious agents, including both respiratory and enteric viruses, as well as a variety of non-viral pathogens, are spread by contaminated hands (World Health Organization, 2009). Hand washing with soap and water has been recommended as one of the most important measures for prevention of dissemination of the spread of viruses, including SARS-CoV-2 (United States Centers for Disease Control & Prevention, 2020a). In healthcare settings, non-compliance with hygiene practices predisposes healthcare workers to the dissemination of nosocomial infections. This is an important lesson, well known in the infection prevention and control community, but perhaps only more recently by the public-at-large. During a pandemic associated with an extremely contagious respiratory (and possibly enteric) virus, such as SARS-CoV-2, the routes of infection spread can be visualized as a cycle from infected persons to non-infected persons directly through contaminated droplets suspended in the air and/or contaminated high-touch environmental surfaces (HITES) and hands directly or indirectly (Fig. 1).

## Survey methodology

This traditional review of the literature was intended to address, at a high level, a number of topics germane to the efficacy of soap and water hand washing as an intervention for limiting the person-to-person spread of SARS-CoV-2. As such, our methods for ascertaining relevant literature were not intended to allow access to all of the literature. In fact, we did not use, at the onset, exclusion criteria for accessing the literature. We simply searched the relevant literature in Google Scholar and PubMed on each of the topics covered in the review (see abstract and the subject headings below). Search topics used therefore included SARS-CoV-2 and coronaviruses; transmission routes; persistence (stability) on surfaces, skin (hands), and human excreta; general relevance to infection prevention and control of hand hygiene and hand hygiene practices; mechanism of action of soap and water washing for reducing contamination of hands; soap formulation components and their functions and potential contributions to pathogen inactivation; the role of antibacterial soaps and potential antimicrobial components in hand soaps; efficacy of hand soap products specifically for inactivating SARS-CoV-2; the concept of contact time and decimal reduction value as this informs duration of hand washing; the role of water temperature in hand washing as an intervention for limiting spread of SARS-CoV-2; the role of hand drying options and preferability of disposable hand towels during an infection outbreak; hand washing with soap and water *vs*. use of hand sanitizing agents. As the review intended to emphasize efficacy specifically for SARS-CoV-2, the search of the literature focused on 2020 and early 2021 papers. The older literature was also searched for many of the topics described above, as papers specific to SARS-CoV-2 were not able to be identified in all cases.

## The patient’s bodily fluids-hites-hands-mucous membrane nexus

Viral spread may also occur indirectly, following deposit of virus on HITES and then conveyed to susceptible tissues, such as mucous membranes (especially, the eyes, nose, and mouth), through the intermediacy of the hand (Fig. 1). This indirect route may be thought of as occurring through a patient’s bodily fluids-HITES-hands-mucous membrane nexus (Ijaz et al., 2020a). Respiratory and enteric viruses, such as influenza viruses, rhinoviruses, noroviruses, rotaviruses, adenoviruses, and coronaviruses, have been reported to persist (remain infectious) on HITES and prototypic surfaces for varying lengths of time (Scott et al., 2020). The persistence of SARS-CoV-2 on various types of surfaces may range from minutes to days (Aboubakr, Sharafeldin & Goyal, 2020; Ijaz et al., 2021b). This is also true for coronaviruses in general (Wolff et al., 2005; Aboubakr, Sharafeldin & Goyal, 2020; Kampf et al., 2020; Ren et al., 2020). Spore-forming bacteria (*e.g*., *Clostridium difficile*) and enteric parasitic cysts/(oo)cysts and ova have shown to survive on HITES under ambient conditions for weeks to months (Scott, Bruning & Ijaz, 2021). Additionally, enteric parasitic ova/(oo)cysts have been recovered from the hands of naturally contaminated Bangladeshi and Indian populations (Ijaz et al., 2013).

Various studies have reported the numbers of infectious units of different pathogens recoverable from contaminated hands of healthcare workers. For example, an investigation by Ehrenkranz & Alfonso (1991) reported that when gloves were not worn by healthcare workers, 15% of nurses working in an isolation unit carried a median of 10,000 colony-forming units of *Staphylococcus aureus* on their hands and 29% of nurses working in a general hospital had a median count of 3,800 colony-forming units. In another study (Daschner, 1989), 21% of doctors and 5% of nurses were found to carry > 1,000 colony-forming units of *S. aureus* on their hands. Casewell & Phillips (1977) found that nurses could contaminate their hands with 100–1,000 colony-forming units of *Klebsiella* spp. (Beggs, Shepherd & Kerr, 2008; World Health Organization, 2009; Awoke et al., 2018). Despite such evidence of the potential for healthcare workers to disseminate pathogens *via* contaminated hands, it has been estimated that these professionals practice hand hygiene fewer than half the time that they should (United States Centers for Disease Control & Prevention, 2011). Viruses have found to survive on experimentally contaminated hands, and secondary transmission to clean hands of other persons or HITES has been reported (Barker, Vipond & Bloomfield, 2004; Bidawid et al., 2004; Boone & Gerba, 2007; Winther et al., 2007; Kampf, Löffler & Gastmeier, 2009; Fong et al., 2020). In addition, in laboratory studies SARS-CoV-2 has been shown to survive on swine skin (a surrogate used for human skin, Harbourt et al., 2020) or human skin (Hirose et al., 2020), with a decay half-life of 3.5 to 9.0 h under ambient conditions.

These data inform the need for practicing hand hygiene per the recommendations of public health agencies, especially during the SARS-CoV-2/COVID-19 pandemic. According to the United States Centers for Disease Control & Prevention (2011) and the British Columbia Centre for Disease Control (2020), 80% of common infections are spread by hands, and washing hands at least five times a day has been shown to significantly decrease the frequency of acquiring colds, influenza, and other infections. In addition, hand washing helps prevent the spread of infectious agents to others. According to the United States Centers for Disease Control & Prevention (2020a), “keeping hands clean is one of the most important steps we can take to avoid getting sick and spreading germs to others.” Also, per the CDC (United States Centers for Disease Control & Prevention, 2020a), hand washing:

1. Reduces the number of people who get sick with diarrhea by 23–40%
2. Reduces diarrheal illness in people with weakened immune systems by 58%
3. Reduces respiratory illnesses, like colds, in the general population by 16–21%
4. Reduces absenteeism due to gastrointestinal illness in schoolchildren by 29–57%

The important roles that hands and HITES play in dissemination of pathogens are well established. This is especially true in the case of enteric viruses (enteroviruses, noroviruses, and rotaviruses). Gastrointestinal symptoms have also been reported for MERS-CoV, SARS-CoV, and SARS-CoV-2 (Kwan et al., 2005; United States Centers for Disease Control & Prevention, 2019; Caio et al., 2020; Wang et al., 2021). This indicates a potential for transmission through the oral-fecal route. In fact, the relevance of this route of transmission may have informed the public health agencies in recommending the hand hygiene intervention early on in the SARS-CoV-2/COVID-19 pandemic. There is a need for vigilance in maintaining appropriate hand hygiene practices, including the ancient and simple practice of washing hands with soap and water. With the availability of modern microbicides, disinfectants, sanitizing agents, and sanitizing hand rubs, it might not be clear why hand washing is still being recommended for infection prevention and control of SARS-CoV-2. We attempt to answer this question in the remainder of this article.

## Handwashing with soap and water leads to inactivation and removal of pathogens

**Figure 2:**
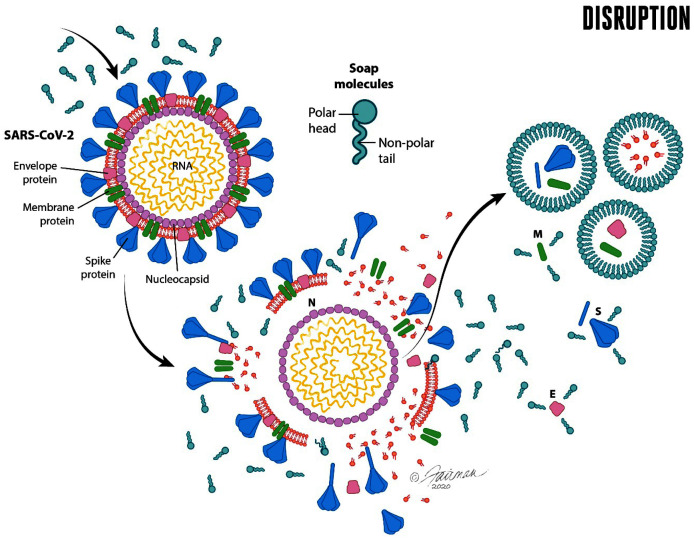
Soap and water remove dirt and other types of organic load, and adherent pathogens (bacteria and bacterial spores, viruses such as SARS-CoV-2, fungi, and enteric parasitic ova and protozoan (oo)cysts) from the skin. Modified from Thordarson (2020).

The use of soap followed by water rinsing is an effective intervention for decontaminating hands contaminated with enveloped viruses, such as SARS-CoV-2. Performed properly, hand washing with soap and water has important orthogonal mechanisms of action relevant to decontamination of hands (Chaudhary et al., 2020). The first of these is mechanical removal of dirt and organic load (such as mucus, sputum, other bodily secretions/excretions) to which virus may have adhered once released from an infected person. The removal function of soap and water is dependent on lathering and mechanical rubbing. Hand washing with soap and water accomplishes the physical removal of pathogens (including enveloped and non-enveloped viruses, bacteria, bacterial spores, and enteric parasitic ova and protozoan parasitic (oo)cysts) adhering to the skin. This physical removal also includes pathogens associated with dirt or organic or inorganic load, known as soil load (Sickbert-Bennett et al., 2005; Ijaz & Rubino, 2008; Grayson et al., 2009; World Health Organization, 2009; Conover & Gibson, 2016; Scott, Bruning & Ijaz, 2021). Soap in aqueous solution forms micelles. The ability of water to dissolve polar (hydrophilic) portions of the dirt or soil load is complemented by the ability of the soap micelles to dissolve the non-polar (hydrophobic) portions of the soil load and lipid-enveloped viruses (Fig. 2).

Once the polar and non-polar components of the dirt, soil load, and associated pathogens have been solvated, each of these may be physically removed through the lathering, rubbing, and water rinsing process. As will be alluded to below, it takes time for the solvation processes to occur, and time to effectively rinse the solvated components off of the hands. Guidance on duration of hand washing for at least 20–30 s (*e.g*., United States Centers for Disease Control & Prevention, 2020a; World Health Organization, 2009) reflects, in part, these realities.

**Figure 3:**
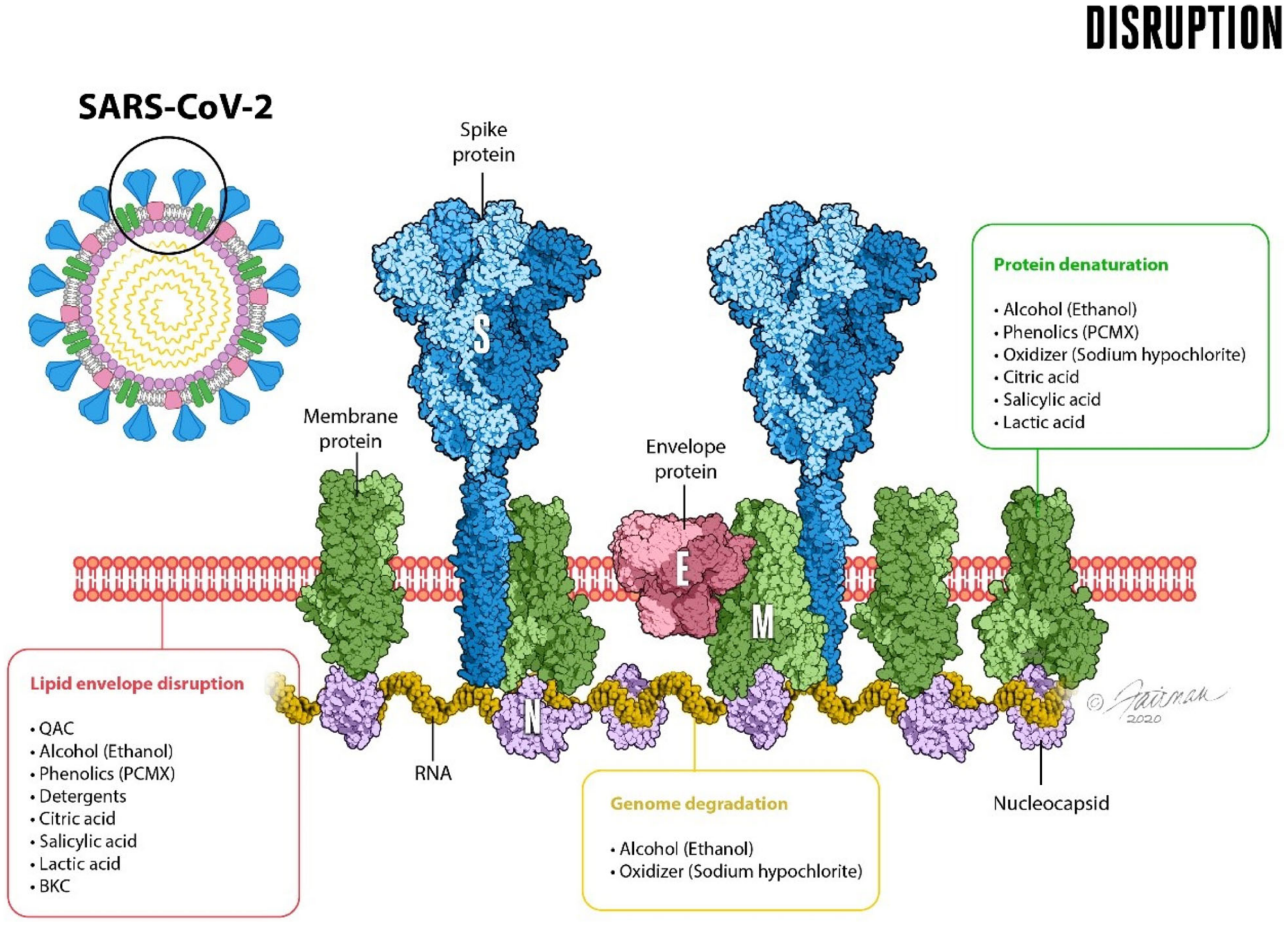
Schematic representation of the enveloped virus SARS-CoV-2 and depiction of the lipid envelope, indicating ultrastructure and mechanisms of action of microbicides, including components of formulated soap (from Ijaz, Nims & McKinney, 2021a). The viral spike (S), envelope (E), and membrane (M) proteins are depicted, as is the nucleocapsid (N). Reprinted from Journal of Hospital Infection Publication title, Vol 112, Ijaz MK, Nims RW, McKinney J, SARS-CoV-2 mutational variants may represent a new challenge to society, but not to the virucidal armamentarium, 121-123, 2021, with permission from Elsevier.

The second orthogonal method of decontamination of hands by soap and water involves inactivation (reduction in infectivity) of enveloped viruses, including SARS-CoV-2. Compared with other microorganisms, enveloped viruses have a very simple structure (Fig. 3). All viral particles consist of genetic material (either DNA or RNA) that is enclosed within a protein structure termed a capsid. In the case of enveloped viruses, such as SARS-CoV-2, the capsid is covered with a lipid envelope derived from the host cell. Embedded in the lipid envelope are viral-encoded glycoproteins that enable the virus to interact with the appropriate host-cell receptor and initiate infection. For SARS-CoV-2, the primary host cell receptor has been reported to be angiotensin-converting enzyme 2 (ACE2) (Yan et al., 2020; Hoffmann et al., 2020).

Such receptor interactions confer specificity (tropism) for infecting the cells of certain animal species, or even certain tissues of a given animal species, as these interactions determine the ability of the virus to enter the cell and to initiate an infection of that cell, as described above.

**Figure 4:**
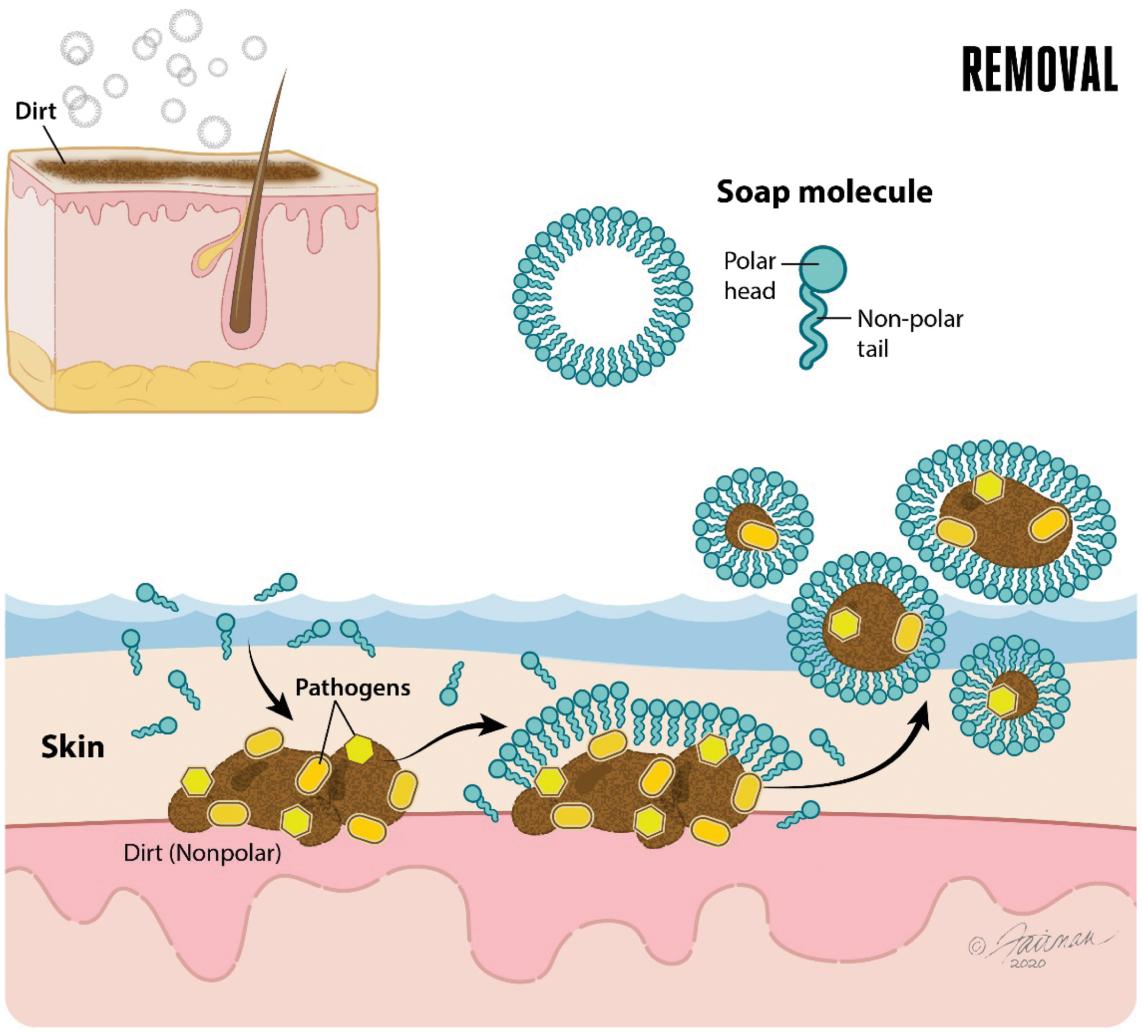
Micellization of envelope components of disrupted viruses, including SARS-CoV-2, by soap and water. The formation of soap micelles occurs with hydrophilic (polar head) portions of the molecule extending outward and the hydrophobic (non-polar tail) portions extending inward. M, membrane protein; E, envelope protein; S, spike protein; N, nucleocapsid protein. Illustrations by: Jennifer Fairman, © 2021, Fairman Studios, LLC.

Here is where the inactivating effects of soap enter the picture. The viral inactivation is primarily the consequence of disruption of the lipid envelope by the soap, as follows. Soap (and other detergents/surfactants) are capable of disrupting the continuity of the viral envelope, essentially through dissolving the envelope within and among the soap micelles (Fig. 4).

When the viral envelope is so disrupted, the host-cell receptor-binding ability of the virus is compromised and the virus can no longer initiate an infection. In this respect, enveloped viruses are actually the most susceptible of microorganisms to chemical inactivation (Ijaz & Rubino, 2008; Ijaz et al., 2020a). The inactivation by soap and water is no exception, and it is indeed fortunate, from a microbicidal susceptibility point of view, that SARS-CoV-2 and many other emerging and re-emerging viruses of public health concern (Ijaz et al., 2020a), are lipid-enveloped. Soaps or detergents (which by definition are surfactants-see below) begin to destabilize the lipid component of the envelope, leading to fragmentation of the lipid bilayer with embedded proteins. There is association of these fragments with surfactant monomers and possible micellization of these fragments, as well as of free proteins (Le Maire, Champeil & Møller, 2000; Simon et al., 2021), as depicted in Fig. 4.

## Synergistic role of soap ingredients in removal and/or inactivation of pathogens

Soaps are sodium or potassium salts of long-chain (≥8 carbons) fatty acids, and are, by definition, surfactants. Surfactants are substances which lower the surface tension of the medium or the interfacial tension of solutions with mixed phases (*e.g*., aqueous/organic). Not all surfactants (surface active agents) are soaps (IUPAC, 1997). The biophysical and biochemical interplay between different soap components contributes to pathogen mobilization and removal, as well as to other characteristics of the formulated soap that include mildness and cleaning efficacy (removal of dirt, oils, and other bodily and environmental pollutants).

### Surfactants

The primary non-water component of bar soaps and liquid hand-and body-wash formulations is the surfactant. The selection of surfactants during formulation development impacts formula cost, formula stability, consumer experience in lathering and rinsing and skin mildness, as well as pathogen inactivation and removal efficacy. Often, skin mildness is achieved through blending various types of surfactants, such as an anionic with an amphoteric surfactant. This helps offset the harshness of the anionic surfactant and subsequent emulsification of lipids from the skin (Hall-Manning et al., 1998). While it might be expected that a stronger and more aggressive cleanser might help remove pathogens from the skin, in fact a balance with skin mildness must be struck so as not to dry out the skin or strip away skin lipids or healthy commensal microbiota. Surfactant type and level have been strongly linked to pathogen inactivation efficacy of soaps (Shafa & Salton, 1960; Al-Adham, Haddadin & Collier, 2013; Jensen, Rogers & Schaffner, 2017; Simon et al., 2021). Anionic surfactants, are known to be effective against bacteria, and this is enhanced at higher surfactant levels. Shafa & Salton (1960) demonstrated that SDS (sodium dodecyl sulfate) caused disaggregation of the bacterial cell wall at various concentrations and pH. The effect was maximized at a 14-carbon chain length. In another study, a positive correlation between hydrophile/lipophile balance and log_10_ reduction values for bactericidal effect was observed (Jensen, Rogers & Schaffner, 2017). Further, anionic surfactants have demonstrated virucidal activity against enveloped viruses (Piret et al., 2002; Tsujimura et al., 2015). Non-ionic surfactants also possess virucidal efficacy for enveloped viruses (Asculai et al., 1978). Cationic surfactants, or quaternary ammonium compounds (QAC) are well documented as having bactericidal properties, and a more thorough discussion of the actives such as benzalkonium chloride (BKC) may be found later in this review. Although generally robust in bactericidal efficacy at pH > 3.5, these agents display poor detergency and foaming performance (Al-Adham, Haddadin & Collier, 2013). QAC also display limited chemical compatibility with anionic surfactants and may lose efficacy in the presence of nonionic surfactants, due to micellar kinetics (Al-Adham, Haddadin & Collier, 2013).

Generally, surfactants interact with bacteria or viruses in two ways (Chaudhary et al., 2020; Simon et al., 2021). The first is through removal, by suspending and removing the bacteria or virus particles from the skin surface and carrying these away in the rinse water, much as done with particulate soil. This occurs as a result of the hydrophobic tails of the surfactant molecules interacting with the lipophilic cell wall (bacteria) or lipid envelope (enveloped viruses). The pathogens are mobilized from the surface, dispersed in the solution by the surfactant, and subsequently are removed with the rinse water. This process may be assisted by the removal of dead skin cells with each wash. Secondly, surfactants solubilize the lipophilic cell wall of bacteria and the lipid envelopes of enveloped viruses and disrupt these structures. This disruption allows the contents of the bacteria/virus to be released or may allow nucleases/proteases or additional formulation components to enter the bacterium/viral capsid. This process may be hindered by the presence of cholesterol in the cell membrane (Riske et al., 2017). The cell wall composition may also influence the rate at which the process of lipid disruption occurs (Lete et al., 2019). In the case of Gram-positive bacteria, there is no outer membrane. Instead, a thick peptidoglycan layer exists, which is composed of peptidoglycan and teichoic acid. This layer does not present a barrier for surfactant to pass into the bacterium (Nillian et al., 2016). Disruption of this layer, however, allows cellular content to be released, leading to enhanced microbicidal activity. In the case of non-enveloped viruses, disrupting the capsid protein presents a greater challenge. Recent studies on norovirus virus-like particles indicate that anionic and cationic surfactants may influence particle aggregation by altering the surface charge of the capsids (Mertens & Velev, 2015). It has been noted that concentrations above the critical micelle concentration are needed to cause disassembly of the capsid proteins. Non-ionic surfactants have been found to have little effect on virus-like particles in suspension (Mertens & Velev, 2015).

While it is apparent from the above that the surfactant component of soaps is a major contributor to bactericidal and virucidal efficacy, the potential for interaction of the surfactants with other components of soaps is important to consider. For instance, the interactions and chemical compatibilities between the surfactants and any antimicrobial actives used in the soap formulation must be evaluated (Le Maire, Champeil & Møller, 2000). Surfactants may emulsify lipophilic actives, keeping them contained within the interior of micelles and, therefore, less accessible to pathogens. Non-ionic surfactants, which are more capable of emulsifying lipophilic materials, fall into this category (Shapiro, 2020). Conversely, synergies may be realized when combining certain surfactants and microbicidal actives. As an example, acid plus anionic surfactant sanitizing systems are commonly employed for cleaning and sanitization in the food industry, though their efficacy is dependent on relatively low pH (Richter & Cords, 2001).

### Bar soaps

Bar soaps have salts of alkyl carboxylates of varying chain lengths of -CH_2_-groups (in the range of eight to 18). The actual chain length distribution in a soap depends upon the source oil from which the soap was derived. For example, soaps derived from coconut oil generally contain more soluble lower chain fractions and the tallow-derived soaps contain higher chain lengths. The lower chain length fractions (≤14 carbons) are more soluble than the higher chain length fractions. The latter, with stearates (18 carbons) and palmitates (16 carbons), act as bar soap structural components (Spitz, 2016). The higher chain length fractions with unsaturation in the chains, such as the oleates (C18:1), also contribute to the soluble surfactant components of the bar soap because these fractions display higher solubilities than their saturated counterparts (Fujiwara et al., 1999). The detergency, microbicidal activity, and virucidal activity of soaps essentially are provided by the soluble surfactants possessing the highest surface activity under use conditions. For a homologous series of alkyl soaps, the surface activity increases with increase in the –CH_2_-group chain length and the solubility decreases with increase in the chain length (Fujiwara et al., 1999). Thus, there exists an optimum chain length that can provide sufficient solubility, with high enough surface activity, to provide detergency and antiviral/antimicrobial benefits. The optimum chain length for surface activity and solubility is, generally, in the range of 12 to 14 carbons (Fujiwara et al., 1999). Even though carboxylates have pKa values around pH 5, their low solubilities and complex phase behaviors result in sufficient amounts of soluble fractions existing only at pH ≥ 10. The solubility can be increased, to some extent, with appropriate choice of the counterions for the soaps. For example, potassium soaps are more soluble than sodium soaps. Similarly, larger organic counterions, such as triethanolamine, can further increase solubility.

Another factor that can affect the solubility of alkyl chains in bar soaps is the temperature of the wash solution. For example, the more surface-active longer chains are more soluble at 40 °C than at 25 °C. It is possible, therefore, to optimize the solubility and surface activity of soaps to maximize the antimicrobial and antiviral viral effects through the appropriate selection of carboxylate chain length combinations, pH, and counterions (Fujiwara et al., 1999). The point to note is that all bar soaps are not the same in terms of performance. Their compositions may be tuned to enhance their performance. In general, higher wash temperature will enhance the microbicidal and virucidal activity of soaps, but the use temperature will be limited by possible impacts on skin mildness and comfort during use, as discussed below.

### Chelators

When formulating rinse-off cleaning products, such as hand soaps and hand wash agents, the use of a chelator or builder is necessary. These agents provide stability to a given product under ambient conditions. In particular, chelators protect formulas from rancidity by abrogating the impacts of metal ions, which may arise during manufacture of the product in steel equipment or may derive from packaging components. In a similar fashion, chelators also bind hardness ions, which may be present in water during consumer use and which can bind to the surfactant and suppress lathering. Due to their three-dimensional structures, chelators are able to bind (sequester) the hardness or metal ions, making the latter unable to react with other chemical components in the soap formulation (Baki & Alexander, 2015).

Furthermore, chelators help protect soap formulations from bacterial growth and contribute to antimicrobial efficacy. This is accomplished mainly through chelation of ions from the pathogen’s cell walls. The cell walls of Gram-negative bacteria contain anionic lipopolysaccharides bridged by divalent cations (Ca^2+^ and Mg^2+^). Networks of metal cations between cell wall teichoic acids, in the case of peptidoglycan layers, can also influence the rigidity and porosity of the cell wall. This structural feature stabilizes the cell wall and provides a barrier to entry of hydrophobic molecules into the bacterium. Chelator-mediated removal of divalent cations from lipopolysaccharide can increase the permeability of the outer membrane to large hydrophobic molecules (Brooks et al., 2007). A recent publication also suggests that divalent cations have a stabilizing role in the structure of viral capsids. For instance, exposure to chelating materials and subsequent heating or cooling steps resulted in externalization of parvoviral DNA (*i.e*., inactivating the virus) without disassembly of the capsid structure (Caliaro et al., 2019). More research in this area is required.

### Glycerin

Glycerin is commonly added to soap formulations to help offset the drying and oil stripping effect of surfactants. This is needed for hand washing, due to the frequency with which washing may be required. Glycerin may represent 1–10% or greater of the overall composition in a formula. Higher levels (15–20%) may also aid in formula preservation, by reducing available water (Jungermann & Sonnag, 1991).

### pH

The pH of a soap formulation is important to the health of the skin being washed. The pH of skin is typically between four and six. A hand wash product ideally should be formulated to ensure the maintenance of skin pH in this range, as the pH of the skin is linked with acidification of the stratum corneum, production of microbicidal lipids, and maintenance of commensal microbiota (Wertz & De Szalay, 2020). Elevation of skin pH has been demonstrated to cause detachment of commensal microbiota (Lambers et al., 2006). An antibacterial intimate wash formulated with lactic acid was recently demonstrated to stabilize the pH of the vulvar skin, while causing no negative impacts on the richness or diversity of the microbiome (Bruning et al., 2020). When formulating with organic acids, the pH of the formulation and the pKa of the acid must be considered. The impact to the surfactant component in the formulation should also be assessed, particularly when amphoteric or zwitterionic surfactants are used. Acidification may influence the charge of the surfactant and the stability of the micelles generated in aqueous solutions.

## Antimicrobial soaps and their active ingredients

As explained above, the primary mechanisms of action for hand washing with soap and water include physical removal of free virus and virus-associated with dirt or other organic load from the skin, and inactivation of remaining enveloped viruses through disruption of their lipid envelopes. Antimicrobial (bactericidal and virucidal) soaps contain additional ingredients, such as benzalkonium chloride, benzethonium chloride, chloroxylenol, citric acid, lactic acid, or salicylic acid. Benzalkonium chloride, chlorhexidine, and chloroxylenol are effective against coronaviruses, in general (Kampf et al., 2020; Dev Kumar et al., 2020; Lin et al., 2020), and SARS-CoV-2, in particular (Chin et al., 2020). A liquid hand wash containing salicylic acid as active ingredient and a bar soap containing chloroxylenol have been reported to inactivate ≥ 3.0 log_10_ of SARS-CoV-2 in 60 s (Ijaz et al., 2020b; 2021b). The addition of such agents to plain soap, in formulating antimicrobial soaps, should therefore add synergistic inactivation of an enveloped virus such as SARS-CoV-2 during hand washing. The inactivation of SARS-CoV-2 removed from the hands during washing and rinsing is an important topic, since the dissemination of infectious virus in waste water streams is undesirable from an infection prevention and control point of view. The potential for infectious SARS-CoV-2 to persist in wastewater streams is not empirically proven, although qPCR-based recovery of SARS-CoV-2 RNA in sewage has been reported (Peccia et al., 2020). This remains a significant knowledge gap that has yet to be closed (Mushi & Shao, 2020). There are data on the persistence of infectious virus in water for other coronaviruses, such as transmissible gastroenteritis virus, mouse hepatitis virus 1, and SARS-CoV (Aboubakr, Sharafeldin & Goyal, 2020), therefore it should be assumed that infectious SARS-CoV-2 may survive for some period of time in waste water.

### Organic acids

Many types of organic acids have been used for preservation of food over the years. Many of these acids are naturally derived and some are Generally Recognized as Safe (GRAS). In addition to being microbicidal, many provide secondary benefits to skin, including exfoliation and moisturization. In the case of microbicidal efficacy, organic acids work through several mechanisms: (a) action of low pH on the cell wall; (b) lowering the internal cytoplasmic pH; (c) chelation of metal ions from the cell wall; and (d) perturbation of membrane function. Organic acids may function by a single mechanism or multiple mechanisms, based on their chemistry. Due to this, combinations of organic acids are often used (Gurtler & Mai, 2014). Lactic acid generally works through disrupting osmotic balance across the bacterial cell wall. Once inside the bacterial cell, the acid permeates the cytoplasm and disrupts cellular functions. The cell expends energy in rebalancing pH, putting significant stress on the cell and resulting in the production of free radicals, which damage cellular structures (Boomsma et al., 2015). In contrast, the microbicidal action of citric acid is thought primarily to be driven by chelation of ions from the cell wall (Gurtler & Mai, 2014). Treatment with acid-based washes triggers uncoating of viral capsids through interactions with capsid proteins, causing the viral genomic material to be exposed to proteases and nucleases. Giranda et al. (1992) described specific rhinoviral proteins which were disordered upon exposure to salicylic acid through this process. The magnitude of disorder was noted to increase as exposure time to the acid was increased, although the minimum times evaluated were on the order of ≥6 min.

Viruses vary with respect to the inactivating effects of low pH (Nims, Zhou & Plavsic, 2017). As a generality, non-enveloped viruses are less susceptible to acidic pH than are enveloped viruses. Enteric viruses (*i.e*., those displaying tropism for the gastrointestinal tract) display lower susceptibility to acidic pH, while rhinoviruses (primarily respiratory viruses) are susceptible to low pH. Even within enveloped viruses, susceptibility to low pH depends on the specific virus under consideration. Significant virucidal effects are typically observed at pH ≤ 4 and may require contact times ≥ 30 min (Nims, Zhou & Plavsic, 2017). Treatment with acid washes have been evaluated against rhinovirus and demonstrated to reduce infectivity of these acid-sensitive picornaviruses following 7 to 10 min contact time (Turner et al., 2004). Organic acid-based hand wash agents which lead to persistent acidification of the skin continue to inactivate rhinoviruses for 2 to 3 h after application (Turner et al., 2004).

### Phenolics

Phenolic actives, such as chloroxylenol (*p*-chloro-*m*-xylenol, PCMX), are some of the few remaining actives on the United States Food and Drug Administration consumer antibacterial hand wash monograph (United States Food & Drug Administration, 2020a) as of the time of writing this article. These have been in use for many years and have been studied thoroughly. While most phenolics are synthetic, salicylic acid and thymol may be naturally derived. Generally, these actives are most effective at acidic or neutral pH or in the undissociated state. Gram-positive bacteria are more sensitive to phenolics than Gram-negative bacteria. Phenolics act first by binding to the cell surface. Once bound, phenolics damage the structural integrity of the cell membrane, disrupting the membrane’s ability to act as a permeability barrier. Intracellular material is then released to the surrounding environment. Interaction of the phenolic with the cytoplasmic contents is the final stage and can produce effects varying from inhibition of certain enzymes to complete coagulation of the cytoplasmic contents. The effect on the bacterium depends on the concentration of the specific phenolic used. Non-enveloped viruses tend to be more resistant to phenolics, and phenolics possess greater ability to inactivate enveloped viruses than non-enveloped viruses. Previous studies have shown that PCMX-based surface disinfectants can inactivate herpes simplex type-1 virus (De Szalay & Diemer, 2021). In addition, PCMX displays efficacy against Ebola virus experimentally deposited on steel carriers (Cutts et al., 2019). Additional efficacy data are displayed in Table 1. In general, the efficacy of phenolics may be adversely impacted when included within surfactant-containing formulations, because of the tendency of phenolics to become partitioned into surfactant micelles.

**Table 1:** Virucidal efficacy of hand hygiene products against HCoV-229E or SARS-CoV-2 in suspension studies*.

Product type	Active ingredient concentration	Temperature (°C)	Contact time (minutes)	Organic load	Log_10_ reduction in infectious titer achieved	
					Alpha-coronavirus	Beta-coronavirus
					HCoV-229E	SARS-CoV-2
Bar soap^†^	PCMX (0.014% w/w)	37 ± 1	0.5, 1	5% FBS	≥3.3	≥3.0, ≥4.1^¶^
Hand sanitizing wipes^‡^	Benzalkonium chloride (013%)	RT	0.25	None	NT	≥2.97
Liquid gel handwash^†^	Salicylic acid (0.025% w/w)	37 ± 1	0.5, 1	5% FBS	≥3.6	≥3.1, ≥3.6^¶^
Foaming handwash^†^	Benzalkonium chloride (0.025% w/w)	37 ± 1	0.5, 1	5% FBS	≥3.3	≥3.4, ≥5.0^¶^
	Salicylic acid (0.023% w/w)	37 ± 1	0.5, 1	5% FBS	≥3.6	≥3.0, ≥3.6^¶^

**Notes:**

* FBS, fetal bovine serum; HCoV-229E, human coronavirus strain 229E; PCMX, *p*-chloro-*m*-xylenol; SARS-CoV-2, severe acute respiratory syndrome coronavirus 2, w/w, weight-to-weight.

† Tested according to ASTM E1052-20 (ASTM International, 2020) at Accuratus Lab Services (HCoV-229E) or Microbac Laboratories (SARS-CoV-2); data are from Ijaz et al., 2020b; 2021b.

‡ Tested in suspension method within the limit of detection. Data are from Ogilvie et al. (2020).

¶ Evaluated at an 0.5 min contact time.

RT = room temperature.

### Ethanol

Ethanol may be derived from natural or synthetic sources. It is widely used for cosmetic, drug, and surgical applications, including hand sanitization. Concentrations of 10% volume/volume (v/v) have been reported to be bacteriostatic. Concentrations ≥ 30% v/v are bactericidal, depending on exposure time (Kampf & Arbogast, 2021). Generally, ethanol is effective against enveloped viruses when used at an appropriate concentration. Non-enveloped viruses are less susceptible to inactivation, though ethanol levels of 70% v/v have been shown to inactivate the picornaviruses hepatitis A virus and poliovirus type 1, given sufficient exposure time (Ali et al., 2001). More recent studies demonstrate that the addition of acid provides greater efficacy at contact times of less than 60 s (Kampf & Arbogast, 2021). Ethanol attacks bacteria through protein denaturation or coagulation of the cell wall, the cytoplasmic membrane, and cytoplasmic proteins. Coagulation of enzymes leads to loss of cellular functions (Kampf & Arbogast, 2021). Protein denaturation by ethanol is not as efficient in the absence of water. This may explain why absolute ethanol is less bactericidal than mixtures of water and ethanol (Ali et al., 2001). As ethanol can dry the skin, skin moisturizers are recommended in formulations containing ethanol to help prevent drying. These might include glycerin, glycols, oils, or other polymeric ingredients.

In formulating hand sanitizers with alcohols, the appropriate type (from a regulatory point of view) must be selected for the application and targeted region of sale. Typically, this is ethanol or isopropanol. The regulatory guidance will often advise on the required active level and purity of the raw material that must be used in the final formula. Contaminants, such as methanol or 1-propanol, should be avoided, as these may pose safety risks to consumers (United States Food & Drug Administration, 2020b). If alcohols are to be combined, the impact to product flammability and to shipping and handling requirements should be assessed. Further, it is important to evaluate whether any denaturants are specified for the regulatory regions in which the product is intended to be manufactured or sold.

### Quaternary ammonium compounds (QAC)

Within the United States Food and Drug Administration consumer antibacterial hand wash monograph (United States Food & Drug Administration, 2020a), two types of QAC remain available for use as microbicidal actives, benzalkonium chloride (BKC) and benzethonium chloride (BTC). Of the many types of QAC, BKC is fairly simple, with a 12-to 18-carbon alkyl chain hydrophobic tail. The cationic charge and the surfactant-like properties derived from the alkyl chain make these QAC effective against bacterial cell walls and viral lipid envelopes. A thorough review of the various QAC structure types may be found in Falk (2019). As a generality, QAC with enhanced lipophilicities provide better efficacies, and these include QAC having chain lengths of 12 to 16 carbons (Merianos & McDonnell, 2001). The mode of action of QAC has been described in detail (Merianos & McDonnell, 2001). As the surface of bacteria is usually negatively charged, the cationic head group of the QAC is adsorbed onto the surface of the bacteria. Once adsorbed, the QAC diffuses through the cell wall and binds to the cytoplasmic membrane, causing disruption. The cytoplasmic contents, including potassium (K^+^) ions, are released, ultimately causing the death of the cell. The efficacies of QAC have been studied extensively. Gram-positive bacteria are more susceptible than Gram-negative bacteria. QAC also have displayed efficacy against enveloped viruses but not non-enveloped viruses (Brooks et al., 2007). A recent study by Romanowski et al. (2019) has shown that BKC displays efficacy against adenovirus (a non-enveloped virus). Schrank, Minbiole & Wuest (2020) have suggested that additional studies on the efficacy of QAC against viruses are required, noting that in their review of available data that care should be taken to standardize concentrations used, conditions tested, and reporting of data.

### Chlorhexidine gluconate (CHG)

Chlorhexidine gluconate is another type of cationic active which has been used in personal care products, as well as surgical hand cleansers, surgical preparations, and oral rinses. Its mode of action is very similar to that of a QAC. This active ingredient displays broad-spectrum microbicidal activity, although it has been reported that it is less effective against Gram-negative bacteria (Crabtree, Pelletier & Pruett, 2001). It is also moderately effective against non-enveloped viruses (Parhar et al., 2020; Yoon et al., 2020). This ingredient has been noted to persist on the skin following application, providing continuing microbicidal efficacy for up to 5 days (Crabtree, Pelletier & Pruett, 2001). For formulations intended for skin, additional clinical testing should be conducted, as sensitivity of skin and mucosal membranes (ototoxicity) have been reported.

## Efficacy of plain and formulated soap for inactivating sars-cov-2 and other coronaviruses

To summarize the contributions of different components to the performance of plain and formulated soaps, efficacy must be achieved through a balance between microbicidal/virucidal efficacy and compatibility with the skin as a barrier and a critical component of defense against pathogens. This is of particular importance recently, as consumers wash and sanitize their hands more frequently as a response to the SARS-CoV-2 associated COVID-19 pandemic. Efficacy can often be improved through combinations of many of the components described above, including surfactants, chelators, pH adjusting agents, and microbicidal active ingredients which may potentially attack pathogens through multiple mechanisms of action. The finished soap product should also ensure that the skin barrier is protected, and that the pH of the skin is not adversely impacted. An assessment of the impact of the formula on the microbiota of the skin is also strongly recommended, as the microbial community may play an important role in skin’s ability to defend against pathogens.

In the above sections, we have described the mechanisms underlying inactivation of pathogens by soap and water washing. What evidence do we have of the actual virucidal efficacy of hand soaps against SARS-CoV-2 or other coronaviruses? In fact, there is a relative paucity of empirical data on this topic, as noted also by Mukherjee et al. (2021). This is true, despite the recommendations from the infection prevention and control community regarding the importance of hand hygiene with soap and water during the SARS-CoV-2 pandemic. More virucidal efficacy data for plain and formulated soaps need to be generated for SARS-CoV-2 and other enveloped viruses.

Chin et al. (2020) evaluated the efficacy of a diluted (1:49) hand soap solution in a suspension inactivation study and found that 15 or 30-min contact times each led to ≥3.0 log_10_ inactivation of SARS-CoV-2, while a 5-min exposure did not completely inactivate the virus. Unfortunately, in the Chin et al. (2020) paper, full experimental details regarding the testing performed were not conveyed, especially the test method itself, the soap composition tested, and the techniques used for neutralization for mitigation of potential cytotoxicity from the soap prior to determining residual virus. These factors and others may have impacted the determined efficacy. Mukherjee et al. (2021) evaluated the efficacy of a bar soap for inactivating SARS-CoV-2. The soap was tested using the ASTM E1052-11 methodology (ASTM International, 2011) as an 8% w/v solution at 40 °C with 20 s contact time. Three different bar soaps of varying total fatty matter content each resulted in complete (≥3 to ≥4 log_10_ inactivation). Ijaz et al. (2020b), Ijaz et al. (2021b) tested bar soap containing *p*-chloro-*m*-xylenol (tested at 0.014% w/w), reporting ≥ 4.1 log_10_ inactivation of SARS-CoV-2 after a 30-s contact time, following the ASTM E1052-20 methodology (ASTM International, 2020) in the presence of a 5% fetal bovine serum organic load. Using the same methodology, these authors also evaluated a liquid handwash and a foaming hand wash containing salicylic acid (tested at ~0.025% final concentration) and a foaming hand wash containing benzalkonium chloride (tested at ~0.025% final concentration). These hand wash products (Table 1) caused ≥3.0 to ≥5.0 log_10_ inactivation of SARS-CoV-2 and ≥3.3 log_10_ to ≥3.6 log_10_ inactivation of human coronavirus 229E after a 30 to 60-s contact time. In each case listed in Table 1, the inactivation of the coronavirus by the soap product was complete, meaning that no infectious virus could be recovered following exposure to the product. The log_10_ reduction values shown were a function of the input viral titers and the limit of detection of the titration method used. The latter is impacted by the toxicity of the residual soap solution to the cells used for titrating the residual virus.

## Why is adherence to the recommended contact time so critical?

As explained in detail above, the criticality of the recommended (20-s) handwash time is based both upon the removal and the inactivation functions of hand washing with soap and water. In the case of removal, it takes time to generate the lather required to solvate fats and oils, and to mobilize dirt from the skin surface (Jensen et al., 2017; Chaudhary et al., 2020). Once a pathogen associated with such bodily fluids or dirt has been solvated, it takes time to remove the solvated and mobilized (non-adherent) dirt, organic load, and virus from the skin through rinsing. Jensen et al. (2017) recommended a minimum lather time of 10 s, based on their studies with *Escherichia coli*. Rinsing time (~10 s) would then need to be added on to this lathering time.

The concept of the decimal reduction (*D*) value is important for considering contact time with soap for achieving viral inactivation. In other words, the inactivation of SARS-CoV-2 by soap is expected to be time-dependent. The time kinetics of inactivation of SARS-CoV-2 may or may not be first-order (*i.e*., log_10_ reduction in infectious virus may not be linear with respect to time). This does not change the fact that a certain duration of time (lathering time, contact time, rinsing time) is required to achieve a desired extent of inactivation. Unfortunately, this topic has not been addressed empirically, to our knowledge, with the same rigor as has been applied to microbicides intended for application to inanimate hard or soft surfaces (fomites). That is, systematic determination of *D* values for inactivation of different viruses by soap and water were not found during our search of the literature, and this represents an area for future research. Lipid envelope-disrupting hand wash agents, as a class, appear to display efficacy after relatively short contact times. For instance, Ogilvie et al. (2020) found that QAC-based products inactivated SAR-CoV-2 in various soil loads within 15 s. Lavelle et al. (1989) showed that exposure to a hand wash product containing the phenolic *p*-chloro-*m*-xylenol caused concentration-dependent inactivation of human immunodeficiency virus (*Retroviridae*) suspended within a blood matrix within 30 s. The inactivation of Ebola virus Makona variant (*Filoviridae*) within a tripartite soil load (bovine serum albumin, tryptone, and bovine mucin) by a liquid hand wash agent also displayed concentration-dependent inactivation within 20 s contact time (Cutts et al., 2020a). Furthermore, disinfectant pre-impregnated wipes containing activated hydrogen peroxide or QAC have been shown to inactivate Ebola virus Makona variant and vesicular stomatitis virus (*Rhabdoviridae*) after a 5-s contact time (Cutts et al., 2020b).

## Water temperature should not contribute to sars-cov-2 inactivation or removal during hand washing with soap

Viruses are susceptible to thermal inactivation. As a generality, the higher the temperature, the less time the virus will remain infectious, and this generality holds for emerging coronaviruses such as SARS-CoV (Duan et al., 2003), MERS-CoV (Leclercq et al., 2014), porcine epidemic diarrhea virus (Quist-Rybachuk, Nauwynck & Kalmar, 2015), and SARS-CoV-2 (Chin et al., 2020). The extent of inactivation by heating is dependent upon a number of factors, the primary ones being the specific virus under evaluation, the temperature, and the time at temperature. The susceptibility of SARS-CoV-2 to inactivation in culture medium was investigated recently by Chin et al. (2020). The experimental design included evaluation of the time kinetics of inactivation at a variety of temperatures (4, 22, 37, 56, and 70 °C), and inactivation was great enough at temperatures ≥ 22 °C to calculate decimal reduction values (*D*, defined as the duration of time required to cause 1 log_10_ reduction in infectious titer).

**Figure 5:**
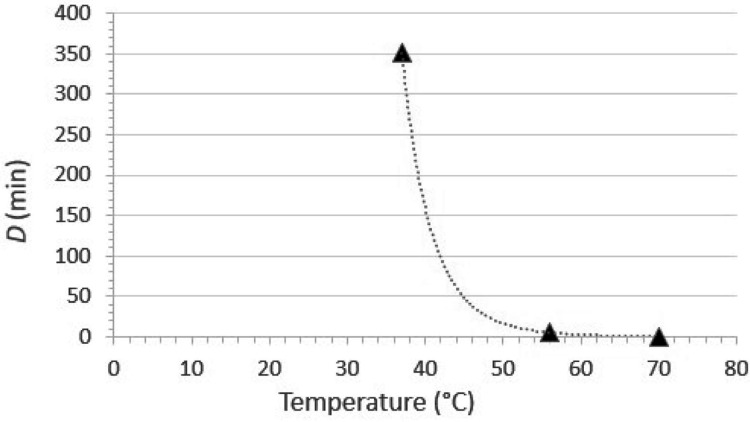
Relationship between *D* (time required to cause 1 log_10_ reduction in infectious titer) and temperature for SARS-CoV-2 thermal inactivation in tissue culture medium (data from Chin et al., 2020). The curve fit is a power function fit, which is the optimal fit for such data (Nims & Plavsic, 2013). The line equation is *D* = 2.70E+18T^−10.13^, where *D* is the decimal reduction value and T is the temperature in °C. The R^2^ value for the line fit is 0.99996.

The temperature susceptibility data mentioned above are only part of the answer. It also is important to consider the water temperature typically used (or expected to be used) for hand washing. According to the U.S. CDC (United States Centers for Disease Control & Prevention, 2020a) “The temperature of the water does not appear to affect pathogen removal; however, warmer water may cause more skin irritation and is more environmentally costly” (the references cited by CDC are Carrico et al. (2013) and Laestadius & Dimberg (2005)). On the other hand, the United States Food & Drug Administration (2005) requires that sinks used for hand washing in retail food settings be capable of supplying water at ≥38 °C (100 °F). The implication of this, though not formally stated, is that the U.S. FDA is recommending that hand washing for retail food preparation staff should be done using water at 38 °C. For the moment, let’s assume that the hand washing is done for the recommended 20 s (United States Centers for Disease Control & Prevention, 2020a) at 38 °C. What contribution to SARS-CoV-2 inactivation can we expect to achieve under these conditions? The first step in answering this question is to plot *D vs*. temperature and then calculate a power fit line. This has been done in Fig. 5, on the basis of the data published by Chin et al. (2020). It is apparent from this plot that at 38 °C, one would have to continue hand washing for over 250 min (the estimate from the fit line is 268 min) to achieve even one log_10_ (90%) inactivation of SARS-CoV-2. At 55 °C, a temperature high enough to cause second-degree burns on skin in 30 s (Carrico et al., 2013), the *D* value for SARS-CoV-2 would be 6.3 min. Less time than this would, again, be essentially useless from an inactivation point of view. Taken together, these data demonstrate that the extent of heat inactivation of SARS-CoV-2 at 38 °C and 20 s contact time is insignificant, and the use of higher temperatures not only would not contribute to viral inactivation, but likely would have the undesirable effect of reducing the barrier properties of the skin.

What about the removal function of hand washing? It has been suggested that higher temperatures might enhance the dissolution of fat by soap and water. This might theoretically improve the dirt-, organic load-, and pathogen-removal function of hand washing. In a practical sense, however, water that is warm enough to be used for hand washing for the recommended period of time (~20 s) should be adequate from a pathogen (including SARS-CoV-2) removal point of view. In fact, Jensen et al. (2017) came to this conclusion in their studies on removal of *Escherichia coli* from hands during hand washing. They reported no significant difference in washing effectiveness at different water temperatures (from 15 to 38 °C). Based on their own data and those of several other investigators cited in their paper, Jensen et al. (2017) questioned the relevance of a required hand wash temperature of 38 °C, suggesting that this should be reconsidered. This conclusion was reached also by Michaels et al. (2002), using *E. coli* as the challenge microbe. Finally, the position of the WHO (World Health Organization, 2009) on this subject is that “Apart from the issue of skin tolerance and level of comfort, water temperature does not appear to be a critical factor for microbial removal from hands being washed. In contrast, in a study comparing water temperatures of 4, 20 and 40 °C, warmer temperatures have been shown to be very significantly associated with skin irritation. The use of very hot water for handwashing should therefore be avoided as it increases the likelihood of skin damage.” The reference cited for the skin irritation concern in the above WHO statement is Berardesca et al. (1995). From the above, it appears clear that the temperature of the water used for hand washing should be dictated by user comfort and not by concerns over impacts on pathogen removal or inactivation.

Even at temperatures deemed to be comfortable and to have less potential for skin irritation, the increased frequency of hand washing now being recommended in response to the SARS-CoV-2-associated COVID-19 pandemic might lead to some irritation of the skin in certain persons. According to Beiu et al. (2020) “Adverse dermatologic effects, such as excessive skin dryness or even contact dermatitis (particularly the irritant subtype and, to a lesser extent, the allergic subtype), can occur, especially in individuals with a history of atopic dermatitis. These skin conditions are perfectly manageable, and applying a moisturizer immediately after washing hands or after using a portable hand sanitizer is the cornerstone in preventing the development of eczematous changes in the hands. In the current global context, the potential occurrence of these dermatological adverse events should in no way cause people to deviate from strict hand hygiene rules.”

## Drying of hands following hand washing

Hand drying is an essential component of effective hand washing that has not received as much attention as the washing process itself (Jumaa, 2005). It is known that the transmission of microorganisms is more effective in wet environments than in dry environments (Patrick, Findon & Miller, 1997). Drying of hands using touch-free electronic hot-air dryers has been touted, due to the fact that (in some cases) there is no need to touch a potentially contaminated surface to engage the drying action. There also are environmental sustainability reasons for advocating the use of such devices. While it might appear that there is less potential for skin abrasion when using hot-air drying devices, compared to the possible friction caused by use of a cloth or paper towel, use of hot-air dryers also may lead to dry, rough, and red hands (Huang, Ma & Stack, 2012). The proper use of a clean paper towel (patting the surface of the skin rather than rubbing it) can contribute to the removal function of hand washing, and does so in a manner which leads to less contamination of the washroom environment (Huang, Ma & Stack, 2012). This is because hot-air hand dryers can disperse pathogens into the air if the hand washing itself has been improperly practiced (Jumaa, 2005; Huang, Ma & Stack, 2012; Kimmitt & Redway, 2015; Del Carmen Huesca-Espitia et al., 2018; Suen et al., 2019; Gammon & Hunt, 2019). The potential for dispersion of infectious SARS-CoV-2 in restrooms by hot-air dryers has not been determined empirically. This, therefore, remains a research gap that needs to be resolved.

## The roles of soap and water *vs*. hand sanitizers for sars-cov-2 decontamination

Hand sanitizers are an important hand hygiene intervention for skin contaminated with an enveloped virus such as SARS-CoV-2. Sanitizers are packaged in units that can be carried easily and therefore are able to be used in high-risk situations (*e.g*., following contact with high-touch environmental surfaces in public places) where the opportunity to wash hands with soap and water is not always possible. In addition, alcohol-based hand sanitizers appear to have less potential for causing skin irritation than do soaps (Gupta & Lipner, 2020; Rundle et al., 2020). One needs to be cautious, however, particularly with repeated use of hand washing with soap immediately before or after using an alcohol-based product under healthcare settings, as this practice could lead to dermatitis (World Health Organization, 2009). Therefore, we recommend that hand sanitizers not be used when soap and water hand wash facilities are available.

Alcohol-based hand sanitizers were found by Grayson et al. (2009) to be more effective than hand washing in reducing human influenza A virus on human hands, though both interventions were found to be effective. For disinfection of hands contaminated with SARS-CoV-2, a variety of types of hand sanitizers should be effective. These include alcohol-based sanitizers (≥60% ethanol or ≥70% isopropanol content is recommended per the U.S. CDC) (United States Centers for Disease Control & Prevention, 2020b). In general, lower concentrations of alcohols (~60%) are effective for inactivation of enveloped viruses, while higher concentrations (70% to 80%) are required for inactivation of non-enveloped viruses, such as hepatitis A and poliovirus (World Health Organization, 2009).

Golin, Choi & Ghahary (2020) have reviewed the efficacy of a variety of hand sanitizers against coronaviruses. These authors also reviewed the relatively limited empirical data obtained from studies directly comparing the efficacy of hand sanitizers *vs*. hand soaps for inactivating enveloped and non-enveloped viruses (Steinmann et al., 2012; Tuladhar et al., 2015). Steinmann et al. (2012) evaluated virucidal efficacy against enveloped viruses (vaccinia virus and bovine viral diarrhea virus) and non-enveloped viruses (poliovirus, adenovirus, feline calicivirus, and murine norovirus), comparing alcohol-based hand sanitizers with antimicrobial soaps in suspension testing and the fingerpad test with 30 s contact time. In the suspension testing method, the sanitizers were effective against all viruses, whereas the soaps were effective against the enveloped viruses only. In the fingerpad test, a povidone-iodine-containing soap was superior to the sanitizers, while the other soaps (containing chlorhexidine or triclosan) displayed less activity. In the Tuladhar et al. (2015) study, the virucidal efficacy of a propanol-based disinfectant against the non-enveloped murine norovirus was compared with that of soap and water. Soap and water washing was found to be superior to the propanol-based sanitizer, causing complete (≥3.0 ± 0.4 log_10_) inactivation within 30 s, while inactivation by the alcohol-based sanitizer was incomplete and variable (2.8 ± 1.5 log_10_) and required greater contact time (3 min).

The efficacies, for inactivating SARS-CoV-2, of three bar soaps and three alcohol-based hand sanitizers were compared in Mukherjee et al. (2021). Each resulted in complete (≥3 to ≥4 log_10_ inactivation), though the test conditions were not the same. For instance, the bar soaps were tested as an 8% solution at 40 °C for 20 s, while the hand sanitizers were tested undiluted (as supplied), at 20 °C, for 10 or 15 s. Wolfe et al. (2017) compared the virucidal efficacy of soap and water *vs*. an ethanol-based hand sanitizer against the enveloped bacteriophage Phi6 (used as a surrogate for the Ebola virus) spiked onto human hands in the presence or absence of a soil load. In the absence of a soil load, the reductions in phage titer obtained following soap and water washing *vs*. use of the ethanol-based hand sanitizer were approximately equivalent (~2.5 log_10_), while in the presence of a soil load, the efficacy of soap and water was superior (3.7 log_10_ *vs*. ~2.5 log_10_ for the hand sanitizer).

The comparison data mentioned above suggest that both hand sanitizers and soap and water display virucidal efficacy for enveloped viruses over short contact times (30 s). Hand washing with soap and water is recommended by the U.S. CDC (United States Centers for Disease Control & Prevention, 2020c) and the WHO (World Health Organization, 2009) when possible, since this hygiene practice achieves pathogen reduction both through removal and inactivation mechanisms, as alluded to above. This is in agreement with findings reported by Foddai, Grant & Dean (2016). In contrast, an alcohol hand rub (ABHR) or other type of hand sanitizer would be expected to inactivate but not necessarily remove infectious virus from the hands. As is the case for hand washing, the effectiveness of hand sanitizers is dependent on the contact time the active ingredient is in contact with the skin, and on the thoroughness of application of the sanitizer to all parts of the hand. According to the U.S. CDC (United States Centers for Disease Control & Prevention, 2020a):

- Alcohol-based hand sanitizers can quickly reduce the number of microbes on hands in some situations, but sanitizers do not eliminate all types of germs.
- Hand sanitizers may not be as effective when hands are visibly dirty or greasy.
- If soap and water are not available, use an alcohol-based hand sanitizer that contains at least 60% alcohol.
- When using hand sanitizer, apply the product to the palm of one hand (read the label to learn the correct amount) and rub the product all over the surfaces of your hands until your hands are dry.

## Discussion

The recent outbreak of SARS-CoV-2 and its associated disease (COVID-19) emphatically has brought to the public’s attention the need for hand hygiene for interrupting the dissemination of the virus and for providing personal protection from becoming infected. We have attempted in this article to provide evidence for the unique utility of the relatively ancient practice of hand washing with soap and water for infection prevention and control during the current SARS-CoV-2/COVID-19 pandemic. The lessons learned from this exercise should be applicable to future outbreaks involving enveloped viruses, as the susceptibility of enveloped viruses is similar, whether one is considering the influenza virus, SARS-CoV-2, or the Ebola virus (Ijaz & Rubino, 2008; Ijaz et al., 2020a). In addition, there are a number of categories of pathogens which exhibit similar susceptibilities to chemical microbicides such as detergents and alcohols. These include vegetative bacteria, yeasts and non-filamentous fungi.

Hand washing is an important intervention for infection prevention and control of viral infections. This is due to the fact that infectious viruses, whether displaying tropism primarily for the upper respiratory tract (*e.g*., rhinoviruses, coronaviruses, and influenza viruses), or for the gastrointestinal tract (*e.g*., reoviruses, enteroviruses, and enteric caliciviruses such as human norovirus) ultimately are spread, in large part, through the intermediacy of the hand (as shown in Fig. 1). While a respiratory infection can be caused by direct inhalation of infectious virus-containing aerosols, dissemination of the virus often involves the intermediacy of the hand. The hands can convey infectious virus from a contaminated surface to the mucous membranes of the eye, nose or mouth, thereby predisposing to the initiation of a new infection in a previously healthy person.

The fecal-oral route, which applies primarily to enteric viruses, also depends on hands as the vehicle for infection of a new host. Recently, Tang et al. (2020) reported an asymptomatic child whose stool sample tested positive for SARS-CoV-2 RT-PCR 17 days after virus exposure. The child was virus positive in stool specimens for at least an additional 9 days. Respiratory tract specimens were negative. Since this report, SARS-CoV-2 has been recovered from stool samples using infectivity assays (Zhang et al., 2020), demonstrating the presence of infectious virus. These results highlight the potential for virus dissemination during changing diapers, and call for practicing appropriate hand and surface hygiene practices by parents of infants. The results also suggest that a combined/paired hygiene approach proposed by Lei et al. (2020) for intervention of pathogens should be applicable to SARS-CoV-2 dissemination from fomites. Additionally, frequent HITES decontamination using surface care disinfectants and wipes, is necessary, particularly when someone is infected with SARS-CoV-2 in a household. This includes such HITES as diaper changing areas, bathroom surfaces, toilet lids, door knobs, etc. Appropriate toilet and kitchen hygiene also must be practiced (Meng et al., 2020).

Why do the hands play such an important role in spread of viruses (and other pathogens)? By habit, we frequently touch our face, including mucous membranes of the nose, eye, mouth. For instance, it has reported (Kwok, Gralton & McLaws, 2015) that we touch our face on an average 23 times per hour (44% touching of a mucous membrane and 56% touching of non-mucosal areas). The mucous membranes touched include the mouth (36%), nose (31%), eyes (27%), and all combined (6%) (Kwok, Gralton & McLaws, 2015). This frequent touching of facial mucous membranes is believed to be taking place in the case of COVID-19, hence the emphasis on hand hygiene by the infection control communities and public health agencies.

The limitations of this review include the following. As the SARS-CoV-2 pandemic has been a relatively recent occurrence, literature addressing certain topics may be limited. This included mechanisms of action of soap and water for removal and inactivation specifically of SARS-CoV-2, efficacy of plain soaps for inactivation or removal specifically of SARS-CoV-2, and contribution of soap components (including microbicidal components) to inactivation or removal specifically of SARS-CoV-2. In some cases, we have had to discuss information for other enveloped viruses or for vegetative bacteria or parasites, recognizing that efficacy for the latter is generally informative for SARS-CoV-2 as well. Finally, there are little clinical data to support the utility of hand washing in preventing acquisition of SARS-CoV-2 or other coronaviral infections (*e.g*., Fung & Cairncross, 2006). Several trials focusing on hand washing have been carried out (Luby et al., 2005; Cole et al., 2012). A very recent literature review revealed that hand-washing reduces diarrhea episodes in child day-care centers in high-income countries and among communities living in low-and middle-income countries by ~30%. The reviewed trials did not provide evidence of the long-term impact of the interventions, however (Ejemot-Nwadiaro et al., 2021) and this topic deserves further investigation. In particular, we are not aware of clinical trials which have been able to focus specifically upon the benefit of hand washing while ruling out other confounding interventions, such as surface hygiene, mask wearing, and social distancing. Other knowledge gaps have been discussed within the body of this review.

We have attempted to clarify certain questions that might come up regarding the need for hand hygiene, the mechanism of action of hand washing with soap and water, the importance of lathering, rubbing, and soap contact time and water temperature during hand washing, the possible synergistic role of active ingredients formulated into hand soaps, the impact of different hand drying methods that might be used post-hand washing, and the potential role of hand washing along with hand sanitizing agents in infection prevention and control during the current SARS-CoV-2/COVID-19 pandemic. It is hoped that this discussion will not only be useful for infection prevention and control during the current SARS-CoV-2/COVID-19 pandemic, but also during future outbreaks involving emerging/reemerging enveloped viruses and variants such as the alpha, beta, delta, gamma, and lambda variants of SARS-CoV-2 (United States Centers for Disease Control & Prevention, 2021).

## Additional Information and Declarations

### Data Availability

The following information was supplied regarding data availability: This is a review paper and does not have raw data.
